# New Insights into Parvovirus Research

**DOI:** 10.3390/v11111053

**Published:** 2019-11-13

**Authors:** Giorgio Gallinella

**Affiliations:** Department of Pharmacy and Biotechnology University of Bologna, 40138 Bologna, Italy; giorgio.gallinella@unibo.it; Tel.: +39-051-4290900

**Keywords:** parvovirus, structural biology, genetics, oncolytic viruses, antivirals

## Abstract

The family Parvoviridae includes an ample and most diverse collection of viruses. Exploring the biological diversity and the inherent complexity in these apparently simple viruses has been a continuous commitment for the scientific community since their first discovery more than fifty years ago. The Special Issue of ‘Viruses’ dedicated to the ‘New Insights into Parvovirus Research’ aimed at presenting a ‘state of the art’ in many aspects of research in the field, at collecting the newest contributions on unresolved issues, and at presenting new approaches exploiting systemic (-omic) methodologies.

## 1. Introduction

The family Parvoviridae includes an ample and most diverse collection of viruses. According to formal taxonomy [1], viruses in the family are all characterised by a linear ssDNA genome, 5–6 kb, and a small icosahedral capsid, 20–25 nm. The host range comprise both invertebrate and vertebrate hosts, giving rise to the main division into the two subfamilies, respectively Densovirinae and Parvovirinae. Further, different genera are recognised within these subfamilies, based on sequence homologies, reflective of evolutionary relationships. In fact, apart from the more general shared properties, a prominent feature is the ample diversity that can be observed between the members of the different genera regarding structure, genome organization and expression, virus–cell interaction, and impact on the host.

Exploring the biological diversity and the inherent complexity in these apparently simple viruses has been a continuous commitment for the scientific community since their first discovery more than fifty years ago. In addition, the translational implications of research on parvoviruses are relevant. Within the family, some viruses are important human and veterinary pathogens, in need of reliable diagnostic methods and efficient therapeutic, antiviral strategies. Rodent parvoviruses have long been studied not only as model systems, but also as tools for oncolytic therapy. Adeno-associated viruses (AAV) have found their way as sophisticated gene delivery vectors and begin now to display successfully their wide and expanding applicative potential.

The Special Issue of ‘Viruses’ dedicated to the ‘New Insights into Parvovirus Research’ aimed at presenting a ‘state of the art’ in many aspects of research in the field, at collecting the newest contributions on unresolved issues, and at presenting new approaches exploiting systemic (-omic) methodologies. Evolution, structural biology, viral replication, virus–host interaction, pathogenesis and immunity, gene therapy, and viral oncotherapy are a selection of topics that have been addressed in articles collected in this Special Issue.

## 2. The Articles in the Special Issue

Studies on the structural biology of viruses in the family can now collect the results of more than twenty-five years of active research, and about 100 structures resolved at high-resolution level are deposited and available. In this Special Issue, all related information is summarised and discussed in a landmark review paper [2]. Presently, the structures of representative viruses of all the different genera in the family are known, and information on capsid–receptor and capsid–antibodies interactions is accumulating. The importance of knowing at atomic level the topology of the capsid shells of these viruses allows for structure–function studies and has critical implications in several instances. First, when considering the tropism of viruses, this allows studying in detail the virus–host cell interactions, also, as a basis for a rational engineering of viruses as oncolytic agents or transduction vectors. Then, such information allows dissecting the capacity of the immune system to recognise and neutralise viruses, a protective effect against viral diseases but a potential problem when considering the use of virus-derived biologics. The common limitation to these studies is the still-unresolved structure of the VP1 unique region, a fractional moiety in the capsid, with a likely flexible and disordered structure, critical for viral infectivity because of the associated phospholipase activity. A novel enzymatic activity associated with this moiety in AAV2 is now presented in a paper in this collection [3].

Next-generation sequencing (NGS) technologies are now frequently in use and contribute effectively to the discovery of novel viruses in the family, as well as to the definition of their evolutionary relationships. Actually, the family picture of viruses in the family is continuously expanding, and new contributions are presented in this issue too. A most intriguing topic is the growing identification of members in the *Chappaparvovirus* genus, and chiefly the resulting inference of an ancient evolutionary divergence of members of this genus from other genera in the family, based both on genomic and structural comparative data [4]. A taxonomic reassessment of subdivisions in the family will be required to incorporate this novel information, and more upcoming work will certainly elucidate the characteristics of this group of viruses. Additionally, metagenomics sequencing led to the identification of a novel bocavirus in ungulates [5], a chappaparvovirus species in dogs [6] and a densovirus infecting acari [7]. On the other hand, molecular phylodynamics continues to yield valuable information, as in the study on spread and evolution of Carnivore protoparvovirus 1 reconstructed based on NS1/NS2 protein sequences [8]. As is always the case, metagenomics identification of viral sequences in biological samples tells us little about the ecology and potential pathogenetic role of a newly discovered virus, so that epidemiological and correlation studies should be required. In this issue, such a question has been addressed about the recently identified equine parvovirus-hepatitis, raising a concern about its possible transmission through contaminated human and veterinary medical products [9].

Novel technologies also allow a deeper and systemic inspection of the genetics and expression profile of viruses within infected cells. The methylation status of the AAV2 genome is presented in [10], showing a difference between packaged or integrated genomes and an inverse correlation with the capability of integrated genomes to be rescued. Epigenetic regulation of parvoviruses is a topic only rarely addressed, but that possibly would merit more attention when considering the long-term relationship of these viruses to their hosts. The transcription map of *Bombyx mori* bidensovirus has been thoroughly investigated and presented [11]. The transcriptome of Human Bocavirus 1 in polarised airway epithelial cells [12] has been analysed by comprehensive RNAseq, and, in this case, the use of NGS and combination of transcript mapping and quantitative analysis could yield a full insight into viral replication dynamics and expression. The aim now at hand by the application of next generation techniques is to obtain comprehensive paradigms to characterize a viral lifecycle and to interpret the effects of the virus within infected cells, possibly at single-cell level.

The initial phases of virus–cell interaction are a relevant matter of investigation. The interaction of *Junonia coenia* densovirus with the midgut barriers of caterpillars has been analysed in detail, to yield a picture of the initial phases of infection that involve binding to host glycans and later disruption of the peritrophic matrix, as presented in [13]. Concerning the human pathogenic parvovirus B19, its very selective tropism for erythroid progenitor cells critically depends on the presence of a specific receptor for the VP1 unique region, but the subsequent steps that are also critical to the outcome of infection still need to be further characterised. The contribution in this issue [14] provides evidence for a coordinated translocation of viral nucleocapsids and genome uncoating in the nucleus of infected cells.

Regarding translational issues, in addition to the engineering of AAVs as very successful gene transduction vectors, there is a long record of studies on the use of protoparvoviruses as oncolytic agents. Two excellent reviews summarise and address the complex issues [15,16] of the potential of protoparvoviruses as oncolytic viruses, describing their characteristics, the known mechanisms of oncolytic and oncosuppressive activity and in particular, how the interplay and cooperation with the host immune system can affect the control of tumours. After so many years of basic research, the first clinical applications of oncolytic parvovirus begin to yield promising results, this in turn prompting for further research to improve the anticancer profile of these agents. A different experimental approach is presented in [17], where the cytolytic properties of parvovirus B19 NS1 protein towards erythroid progenitor cells are exploited in a context of an Adenovirus-derived transduction vector, to obtain a selective oncolytic activity against megakaryocytic leukaemia cells.

The pathogenetic role and clinical implications of human parvoviruses are addressed in two studies presented in this collection, about the role of human bocaviruses and parvovirus B19. In an observational study [18], a significant association of human bocaviruses to gastroenteritis is reported, thus further expanding their clinical involvement in addition to the established association with respiratory tract infections. In a systematic review and meta-analysis study [19], the significance of the detection of parvovirus B19 genomes in endomyocardial biopsies of patients presenting with myocarditis or dilated cardiomyopathy is discussed. This review should be regarded as a very useful contribution to a long debated and far from settled issue. From such meta-analysis, the conclusion is that the mere detection of viral genomes is just indicative of the propensity of B19 to establish long-term persistence in tissues [20], and that implication as a causative agent in cardiomyopathies needs to be supported by some reliable evidence of biological activity of the virus.

Furthermore, concerning a role of parvovirus B19 in the development of cardiomyopathies, the possible effect of telbivudine in reducing the damage to endothelial progenitor cells caused by the presence of B19 is presented [21]. Telbivudine is an RT-enzyme inhibitor used as an antiviral in treating HBV, thus the protective effect against B19-derived cell damage is an unexpected, cell-targeted, and non-selective activity, a result prompting for further research in this field. More in general, parvovirus B19 is the most pathogenic virus to humans, responsible for a wide spectrum of clinical manifestations whose outcomes depend on a close interaction between the virus and the physiological and immunological condition of the infected individuals. Apart from the need for reliable diagnostics [22], there is an urgent need for antiviral treatments that might go beyond simple supportive or replacement strategies. The review in this issue [23] presents the recent results in this field, that led to the first identification of compounds with antiviral activity against parvovirus B19. These comprise retargeted drugs such as hydroxyurea, broad range antivirals such as cidofovir or its derivative brincidofovir, and novel compounds identified in drug-discovery screening experiments, such as some coumarin or flavonoid derivatives. This research, aimed at closing the gap with respect to antivirals available against other DNA viruses, thus, begins to yield interesting results, prompting for further discoveries meeting clinical needs.

## 3. Conclusions

As a conclusive remark, the collection of articles in this Special Issue devoted to ‘New Insights into Parvovirus Research’ and contributed by distinguished researchers should be regarded as significant for two main reasons, among others. First, some of the articles effectively present a ‘state-of-the-art’ overview in some main topics. Then, many articles show how the application of new methodologies, including but not limited to NGS, can be functional to the establishment of novel and more general paradigms in the field. In the near future, research on parvoviruses will certainly yield more answers to still-unresolved issues.
